# A comparison of combined oral contraceptives containing chlormadinone acetate versus drospirenone for the treatment of acne and dysmenorrhea: a randomized trial

**DOI:** 10.1186/s40834-018-0058-9

**Published:** 2018-04-10

**Authors:** Unnop Jaisamrarn, Somsook Santibenchakul

**Affiliations:** Department of Obstetrics and Gynaecology, Faculty of Medicine, Chulalongkorn University, Bangkok, 10330 Thailand

**Keywords:** Oral contraceptive, Chlormadinone acetate, Drospirenone, Acne, Dysmenorrhea

## Abstract

**Background:**

Oral contraceptives (OCs), aside from contraceptive efficacy, have been widely known for their non-contraceptive benefits. Different progestogens component of the OCs have been shown to improve the skin, hair, menstrual cycle related disorders and dysmenorrhoeic pain. Thus, we compared the efficacy of OCs containing ethinyl estradiol (EE) and chlormadinone acetate (CMA) versus OCs containing EE and drospirenone (DRSP) for the treatment of acne and dysmenorrhea.

**Methods:**

This study was an investigator-blinded, randomized, parallel group study conducted at the Family Planning Clinic, Department of Obstetrics and Gynaecology, Faculty of Medicine, Chulalongkorn University, Bangkok, Thailand. Women aged between 18 and 45 years were randomly assigned into two treatment groups, either EE/CMA at the dosage of 30 mcg/2 mg once daily (OD) or EE/DRSP at the dosage of 30 mcg/3 mg OD. The subjects were evaluated for the OC’s efficacy for the treatment of acne and dysmenorrhea at baseline visit and after 1, 3, and 6 months of treatment.

**Results:**

A total of 180 women were randomized into the study. Each group had 90 women. Baseline characteristics between both groups were comparable. At Month 6, there was a significantly greater reduction of total acne lesion in the EE/CMA group than EE/DRSP (72.2% vs 64.5%; *p* = 0.009). As per the investigator’s global assessment of acne treatment, a higher proportion of the subjects from the EE/CMA group was rated “excellent” than those from the EE/DRSP (75.3% vs 49.4%). More subjects from the EE/CMA group had graded their improvement in acne as “excellent” compared to the EE/DRSP group (66.3% vs 48.3%). A higher proportion of the subjects in the EE/CMA group reported a decrease in dysmenorrhoeic pain as “much decrease” and “decrease”. The absence of dysmenorrhea pain was more frequently found in the EE/CMA group and significantly seen as early as Month 1 also in the EE/CMA group compared to EE/DRSP (47.2% vs 27.3%, respectively). The treatments were generally well-tolerated in both groups. There were no significant differences between both groups for adverse events.

**Conclusions:**

EE/CMA is more effective for the treatment of acne and dysmenorrhea in women with mild to moderate acne vulgaris and dysmenorrhea than EE/DRSP.

**Trial registration:**

Thai Clinical Trial Registry ID: TCTR20170518001 (date of registration: May 17, 2017; retrospectively registered).

## Background

Family planning programs have widely used oral contraceptives (OCs) since their introduction in 1960 [1]. Aside from having high contraceptive efficacy and safety profile, OCs also have non-contraceptive benefits such as for the skin, hair and menstrual cycle related disorders.

Nowadays, there are many OCs one can choose from. The different formulations in estrogen and progestogens make each OC unique. Modern OCs contain less ethinyl estradiol (EE) so that there will be fewer serious adverse effects [2]. As the estrogen component of EE remains the same among different combined OCs, the progestogen component varies according to the different brands of combined OCs available. Different progestogens contribute to the distinctive and unique non-contraceptive benefits. Particular combined OCs containing weak or no androgenic effects or even with anti-androgenic progestogens were of preferred choices for acne treatment. Among these preferred progestogens are chlormadinone acetate (CMA) and drospirenone (DRSP). Furthermore, a systemic review showed positive effects of combined OCs on dysmenorrhea which is a common problem among women who menstruate. It has been shown that certain combined OCs can reduce the frequency and severity of dysmenorrhoeic pain [3].

EE/CMA is a monophasic combined low-dose OC containing CMA 2.0 mg and EE 30 mcg per tablet. In clinical trials, this contraceptive was well tolerated and showed reliable contraceptive efficacy, good cycle control and beneficial anti-androgenic effects on both skin and hair [4, 5]. In a phase III study, there was a 60–70% improvement in acne after six cycles of EE/CMA use [5, 6]. In addition, acne was cured in 90% of the subjects after 12 cycles of EE/CMA.

Another distinct property of EE/CMA is its beneficial effect on dysmenorrhea [7]. In a post marketing surveillance survey, from 1266 subjects, 66% of the subjects were cured of dysmenorrhea and another 14% had reduced symptoms of dysmenorrhea after 12 cycles of EE/CMA use [6]. In another study conducted in 1939 women, they reported a 95% decrease in dysmenorrhea after 4 cycles of EE/CMA use [8].

Another progestogen, DRSP, has anti-androgenic and anti-mineralocorticoid effects but has negligible estrogenic or glucocorticoid activity. EE/DRSP is a monophasic combined OC containing DRSP 3.0 mg and EE 30 mcg per tablet. It has been shown that after 9 treatment cycles of EE/DRSP, the total acne lesion count in women with mild-to-moderate facial acne was reduced by 62.5% [9]. Number of studies have shown that a combination of EE/DRSP reduces the severity of dysmenorrhea with fewer days of dysmenorrhoeic pain and 60–65% of the subjects reported that the severity of dysmenorrhoeic pain has lessened [10–12].

One study was recently conducted in adolescents between the ages 14–19 years to compare the noncontraceptive benefits of EE/CMA and EE/DRSP [13]. The study showed that both EE/CMA and EE/DRSP provided beneficial effects on irregular menstruation, dysmenorrhea, hair and skin disorders but that EE/CMA was shown to be more superior to EE/DRSP [13]. The results may be arguable as it is an observational questionnaire-based study and there may have been some bias in the data collection as well as in the interpretation of the results.

Hence, we compared the efficacy of EE/CMA and EE/DRSP for the treatment of acne and dysmenorrhea among women aged between 18 and 45 years in a randomized controlled trial.

## Methods

### Aim

The aim of this study was to evaluate the efficacy of EE/CMA and EE/DRSP for the treatment of acne and dysmenorrhea as well as the overall clinical effects and safety profiles, including cycle control, blood pressure and body weight.

### Study design

This study was an investigator-blinded, randomized, parallel group study conducted from August 2013 to October 2017 at the Family Planning Clinic, Department of Obstetrics and Gynaecology, Faculty of Medicine, Chulalongkorn University, Bangkok, Thailand. The investigator was unaware of the type of medication being provided to the subjects and assessed facial acne and dysmenorrhea while blinded in this way. The study medications were dispensed by the study nurse. The investigator remained blinded during data analysis. The study was approved by the Institutional Review Board of the Faculty of Medicine, Chulalongkorn University and was conducted in accordance with the ethical principles of the Declaration of Helsinki and the International Conference on Harmonization Good Clinical Practice guidelines.

### Treatment

Subjects were randomly assigned in a 1:1 ratio to each group based on a computer-generated randomization scheme. Subjects randomized to EE/CMA treatment received EE/CMA at the dosage of 30 mcg/2 mg (Belara®; Gedeon Richter Plc.; Budapest, Hungary - Abbott Laboratories Ltd., Bangkok, Thailand) once daily while subjects randomized to EE/DRSP treatment received EE/DRSP at the dosage of 30 mcg/3 mg (Yasmin®; Bayer, Berlin, Germany) once daily. Both groups received the treatment for 21 consecutive days, starting on the first day of the menstruation, followed by 7 days of medication free before starting the next cycle of treatment. The treatment was self-administered for a total of 6 consecutive cycles.

### Subjects

Healthy women between the ages of 18 to 45 years with mild to moderate acne vulgaris and who had dysmenorrhea of any degree of severity were eligible to join the study. Mild acne vulgaris was defined as having comedones as the main type of acne lesion with < 10 papules and pustules. Moderate acne was defined as having 10–40 papules and pustules, 10–40 comedones, and/or mild truncal disease. Subjects who agreed to take the medications as their only treatment for 6 months and signed and dated the informed consent were enrolled into the study. Women who were pregnant, lactating and/or had any hypersensitivity to the study medication were excluded from the study. Subjects with any coexisting medical condition or were taking any concomitant medication that is likely to interfere with the safe administration of EE/CMA or EE/DRSP as per the opinion of the investigator were also excluded from the study. Other exclusion criteria included the use of systemic retinoids within 6 months, systemic antimicrobials within 1 month, topical acne treatment within 2 weeks prior to study enrollment and having a contraindication to OCs.

### Clinical assessments

The subjects were evaluated for the efficacy of the OCs for the treatment of acne and dysmenorrhea at baseline visit and follow-up visits after 1, 3, and 6 months of treatment. The following parameters were recorded at each study visit: body weight, body mass index (BMI), vital signs, acne lesion counts, adverse events, and concomitant medications. Each lesion such as comedones, papules, pustules and nodules was counted individually. The total lesion count was the summation of all lesions. Subjects were provided with a menstrual diary card to record information on treatment compliance and vaginal bleeding. Any unused study medications were returned to the study nurse and documented. The degree of dysmenorrhea severity at each visit was rated by the subjects using a 4-scale assessment (0 = absent/no pain, 1 = mild, 2 = moderate, 3 = severe). Subjects also rated severity of dysmenorrhea compared to the baseline level or before study entry (scale from 1 = very much decrease, 2 = decrease, 3 = no change, 4 = increase, 5 = very much increase). Physical examination and overall assessment questionnaire were performed at the last visit or after 6 months of treatment. The investigator completed a global assessment of the acne treatment using a five-point scale (0 = worse; 1 = no change; 2 = fair; 3 = good; and 4 = excellent). Subjects were also required to complete a self-assessment questionnaire which had three questions to evaluate the treatment efficacy and acceptability: 1. How would you rate your acne improvement since you started this study?; 2. How would you compare this acne treatment with other acne treatment you have used in the past?; and 3. Would you continue to take this treatment if your physician prescribes it?

### Statistical analysis

Continuous variables were presented as mean ± SD. Repeated ANOVA was used to assess the change in acne lesion count from baseline to cycle 6 and between each treatment group, using a significance level of 5%. Fisher’s exact test was used to assess any significant difference in the numbers of adverse events and breakthrough bleeding and spotting. The SPSS version 22 was used to analyze the data.

All randomized subjects who received at least one dose of the study medication and fulfilled the inclusion criteria were included in the intention-to-treat (ITT) analysis. All endpoint assessments were analyzed by ITT.

### Sample size calculation

The sample size for this study was determined by the responder rate from a previous study for the treatment of acne [13]. The proportion of subjects responded to the treatment of acne after 6 cycles of treatment was 0.738 and 0.528 in the EE/CMA group and EE/DRSP group, respectively. The sample size was calculated for a study power of 80% and a significance level of α = 5% (two-sided). A dropout rate of 10% was estimated. Therefore, a total sample size of 180 subjects with 90 subjects per treatment group was needed.

## Results

### Subjects

**Fig. 1:**
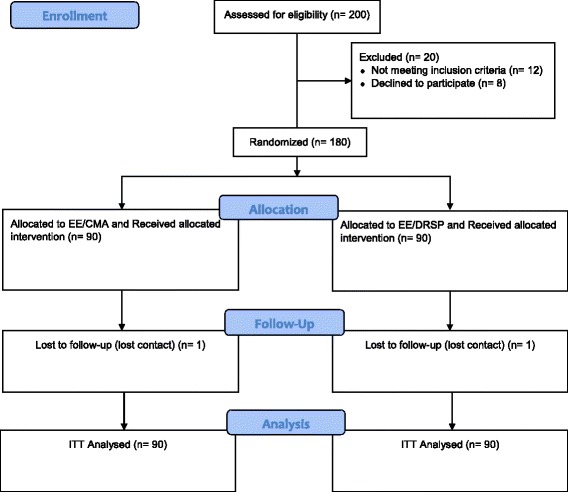
Study Flow

A total of 200 women were screened. Twenty women were excluded from the study because they did not meet the inclusion criteria (*n* = 12) and declined to participate (*n* = 8). A total of 180 women were randomized into the study (Fig. 1). Ninety women were randomized to receive EE/CMA and another 90 women received EE/DRSP. One woman in the EE/CMA group and one woman in the EE/DRSP group were lost to follow up. The age, height, weight, BMI, systolic BP and diastolic BP for both groups were comparable (Table 1).

**Table 1:** Baseline characteristics of subjects who were randomized to receive treatment with either EE/CMA or EE/DRSP

	EE/CMA^a^ (*n* = 90)	EE/DRSP^a^ (*n* = 90)
Age (yrs)	28.5 ± 6.99	27.2 ± 6.05
Height (cm)	158.4 ± 5.79	158.2 ± 4.92
Weight (kg)	56.5 ± 10.02	56.1 ± 8.19
BMI (kg/m^2^)	22.5 ± 3.36	22.4 ± 3.25
Systolic BP (mmhg)	112.1 ± 11.49	110.3 ± 11.39
Diastolic BP (mmhg)	67.2 ± 9.76	67.0 ± 9.35

^a^Continuous variables are presented as mean ± S.D

In terms of treatment compliance, there was no significant difference between the two treatment groups. No missed dose was reported in the majority of subjects in both treatment groups throughout the treatment period. The percentage of subjects who missed one or more doses at each month ranged from 1.1 to 7.8% in the EE/CMA group and from 1.1 to 9.0% in the EE/DRSP group.

### Efficacy

**Fig. 2:**
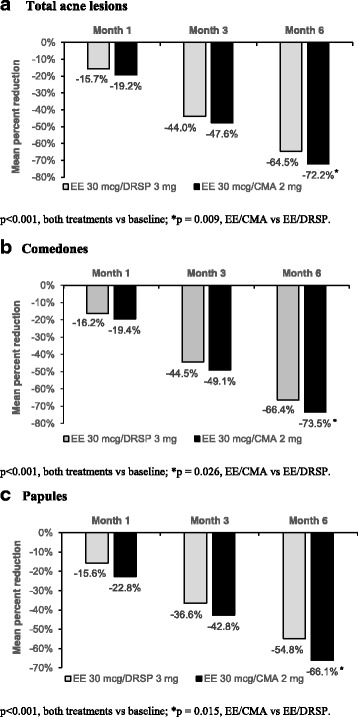
Mean percentage reduction in total acne lesion counts (**a**), comedones (**b**), and papules (**c**) after 1, 3 and 6 months of treatment with EE/CMA or EE/DRSP

There were no significant differences in the number of comedones, papules, pustules/nodules and total acne lesions at baseline between the EE/CMA group and EE/DRSP group (Table 2). Both EE/CMA and EE/DRSP were effective in reducing the acne lesions throughout the 6 months of treatment. However, there was a significantly more reduction of total acne lesion counts at Month 6 of treatment in the EE/CMA group (72.2%) compared to the EE/DRSP (64.5%) group with a treatment difference of 7.69% (*p* = 0.009) as shown in Fig. 2a. Moreover, when considering the different types of acne lesions, there were significantly greater reduction of both comedones and papules from baseline to Month 6 of treatment in the EE/CMA group compared to the EE/DRSP group (Fig. 2b and c).

**Table 2:** Acne lesion counts in subjects after treatment with EE/CMA or EE/DRSP

Testing for Efficacy	EE/CMA^a^ (*n* = 90)				EE/DRSP^a^ (*n* = 90)			
	Baseline	Month 1	Month 3	Month 6	Baseline	Month 1	Month 3	Month 6
	Mean ± S.D.	Mean change from baseline ± S.D.			Mean ± S.D.	Mean change from baseline ± S.D.		
Acne								
Comedones	47.17 ± 22.30	−9.21 ± 1.22	− 23.37 ± 1.67	− 35.00 ± 1.95	42.94 ± 21.08	− 7.02 ± 1.22	− 19.33 ± 1.67	− 28.82 ± 1.95
Papules	14.60 ± 6.63	− 3.34 ± 0.48	−6.29 ± 0.57	− 9.74 ± 0.63	13.67 ± 5.94	−2.16 ± 0.48	−5.06 ± 0.57	−7.57 ± 0.63
Pustules/Nodules	3.1 ± 3.59	−0.23 ± 0.35	−1.55 ± 0.40	−2.38 ± 0.47	3.14 ± 4.29	−0.6 ± 0.35	−2.02 ± 0.397	−2.43 ± 0.47
Total Lesions	64.21 ± 25.09	−12.40 ± 1.35	−30.84 ± 1.75	− 46.78 ± 2.11	59.66 ± 23.44	−9.47 ± 1.35	−26.52 ± 1.75	−38.90 ± 2.11

^a^Continuous variables are presented as mean ± S.D. Counts show changes month by month within treatment groups

**Fig. 3:**
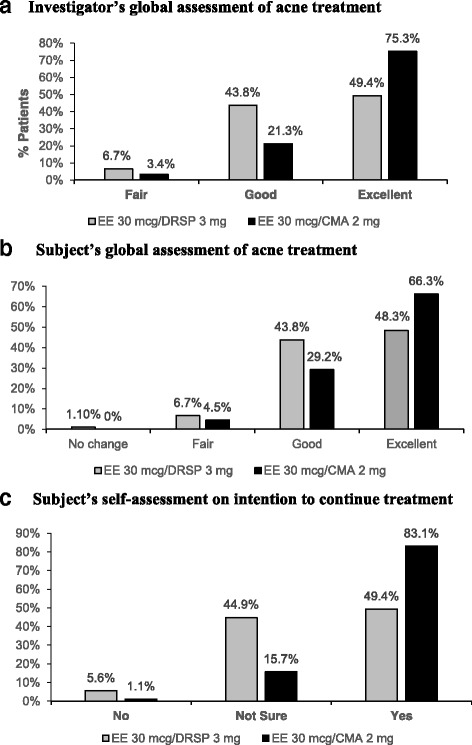
Investigator’s (**a**) and subject’s (**b**) global assessment of acne treatment and subject’s self-assessment on intention to continue treatment (**c**) with EE/CMA or EE/DRSP

Treatment for acne vulgaris was significantly better in the EE/CMA group compared to the EE/DRSP group as per the investigator’s global assessment of acne treatment efficacy (Fig. 3). In the EE/CMA group, 75.3% of the subjects were rated as having an “excellent” response to treatment. On the other hand, only 49.4% in the EE/DRSP group were rated as “excellent” (Fig. 3a). According to the subjects’ self-assessment for acne treatment efficacy, 63.3% of the subjects in the EE/CMA rated the treatment as “excellent” while 48.3% of the subjects in the EE/DRSP rated the treatment as “excellent” (Fig. 3b). Comparing to their previously used contraceptive regimen, 85.4% of the subjects in the EE/CMA reported it being “much better” or “better” whereas 50% of the subjects in the EE/DRSP reported it being “much better” or “better”. When the subjects were asked if they would continue the treatment after the study was completed, the subjects from the EE/CMA group were more willing to continue treatment than the subjects from the EE/DRSP group. In the EE/CMA group, 83.1% of the subjects answered that they would continue the treatment whereas only 49.4% of the subjects in the EE/DRSP would continue the treatment (Fig. 3c).

Change in degree of severity of dysmenorrhoeic pain was assessed at each visit and was compared to the baseline level (Table 3). Throughout the 6-month treatment period, a greater proportion of the subjects in EE/CMA group reported a decrease in dysmenorrhoeic pain as “much decrease” and “decrease” compared to those subjects in the EE/DRSP. There was a significant treatment difference observed at Months 1, 2, and 4 (EE/CMA vs EE/DRSP; *p* = 0.013, *p* = 0.029, *p* = 0.026, respectively). In addition, a greater proportion of the subjects in the EE/CMA group reported an absence of dysmenorrhoeic pain compared to those in the EE/DRSP throughout the treatment period. This difference was significantly seen as early as Month 1 (EE/CMA vs EE/DRSP, 47.2% vs 27.3%) as shown in Table 4. Moreover, a gradual reduction over time in the proportion of subjects requiring medications/treatment for dysmenorrhea were seen in both treatment groups from Month 1 to Month 6 of treatment. Despite that, a greater proportion of subjects who did not require any medications/treatment was observed in the EE/CMA than in the EE/DRSP group throughout the treatment period.

**Table 3:** Severity of dysmenorrhoeic pain during treatment compared to the baseline levels

Visit	Month 1^*^ n (%)		Month 2^†^ *n* (%)		Month 3 *n* (%)		Month 4^#^ *n* (%)		Month 5 *n* (%)		Month 6 *n* (%)	
Severity vs baseline	EE/CMA	EE/DRSP	EE/CMA	EE/DRSP	EE/CMA	EE/DRSP	EE/CMA	EE/DRSP	EE/CMA	EE/DRSP	EE/CMA	EE/DRSP
Much Decrease	49 (55.1)	32 (36.4)	53 (59.6)	41 (46.1)	64 (71.9)	58 (65.2)	82 (92.1)	72 (80.9)	88 (98.9)	85 (95.5)	86 (96.6)	85 (95.5)
Decrease	14 (15.7)	14 (15.9)	29 (32.6)	31 (34.8)	23 (25.8)	26 (29.2)	7 (7.9)	15 (16.9)	1 (1.1)	3 (3.4)	2 (2.2)	4 (4.5)
Not Change	22 (24.7)	40 (45.5)	7 (7.9)	16 (18)	2 (2.2)	3 (3.4)	0 (0)	2 (2.2)	0 (0)	1 (1.1)	1 (1.1)	0 (0)
Increase	4 (4.5)	2 (2.3)	0 (0)	1 (1.1)	0 (0)	2 (2.2)	0 (0)	0 (0)	0 (0)	0 (0)	0 (0)	0 (0)

^*^p = 0.013; ^†^*p* = 0.029; ^#^*p* = 0.026; EE/CMA vs EE/DRSP

**Table 4:** Severity of dysmenorrhoeic pain at each visit

Visit	Baseline *n* (%)		Month 1^*^ n (%)		Month 2^†^ *n* (%)		Month 3 *n* (%)		Month 4^#^ *n* (%)		Month 5 *n* (%)		Month 6^β^ *n* (%)	
Severity of Dysmenor-rhoeic Pain	EE/CMA	EE/DRSP	EE/CMA	EE/DRSP	EE/CMA	EE/DRSP	EE/CMA	EE/DRSP	EE/CMA	EE/DRSP	EE/CMA	EE/DRSP	EE/CMA	EE/DRSP
Absent	0 (0)	0 (0)	42 (47.2)	24 (27.3)	49 (55.1)	37 (41.6)	58 (65.2)	50 (56.2)	74 (84.1)	59 (66.3)	81 (92)	75 (84.3)	84 (95.5)	76 (85.4)
Mild	43 (48.9)	30 (34.5)	30 (33.7)	38 (43.2)	36 (40.4)	36 (40.4)	30 (33.7)	38 (42.7)	14 (15.9)	30 (33.7)	7 (8)	14 (15.7)	3 (3.4)	13 (14.6)
Moderate	45 (51.1)	57 (65.5)	17 (19.1)	26 (29.5)	4 (4.5)	16 (18)	1 (1.1)	1 (1.1)	0 (0)	0 (0)	0 (0)	0 (0)	1 (1.1)	0 (0)

^*^p = 0.008; ^†^*p* = 0.016; ^#^*p* = 0.006; ^β^*p* = 0.026; EE/CMA vs EE/DRSP

### Safety and tolerability

The treatments were generally well-tolerated in both groups (Table 5). Adverse events that frequently occurred in both EE/CMA and EE/DRSP groups were breast pain, dizziness, headache, and nausea. There were no significant differences between both groups for adverse events. The number of episodes of breakthrough bleeding was slightly higher in the EE/DRSP group than in the EE/CMA group at Month 1 (16.9% vs 9%), Month 3 (12.4% vs 9.2%), and Month 6 (2.3% vs 0%) but this was not significantly different between both groups (Table 6). Similarly, there was no significant difference in the incidence of withdrawal bleeding after 1, 3, and 6 months of treatment between both groups (Table 7). In addition, there were no significant changes in body weight, BMI, and blood pressure between baseline and at each study visit during the treatment period for both treatment groups.

**Table 5:** Adverse events after treatment with EE/CMA or EE/DRSP

Adverse event	EE /CMA *n* (%)	EE /DRSP *n* (%)
Breast pain	12 (13.3)	12 (13.3)
Headache	6 (6.7)	9 (10.0)
Nausea	9 (10.0)	8 (8.9)
Dizziness	11 (12.2)	11 (12.2)
Fever	8 (8.9)	3 (3.3)
Flatulence	1 (1.1)	2 (2.2)
Stomachache	2 (2.2)	0 (0.0)
Diarrhea	2 (2.2)	2 (2.2)
Pelvic pain	1 (1.1)	4 (4.4)
Vomiting	2 (2.2)	1 (1.1)
Excessive hungry	4 (4.4)	3 (3.3)

Note: No significant difference between treatment groups

**Table 6:** Incidence of breakthrough bleeding during treatment with EE/CMA or EE/DRSP

	EE /CMA *n* (%)	EE /DRSP *n* (%)
Month 1	8 (9.0)	15 (16.9)
Month 3	8 (9.2)	11 (12.4)
Month 6	0 (0.0)	2 (2.3)

Note: No significant difference between treatment groups

**Table 7:** Incidence of withdrawal bleeding after treatment with EE/CMA or EE/DRSP

	EE /CMA n (%)	EE /DRSP n (%)
Month 1	82 (91.1)	82 (93.2)
Month 3	84 (94.4)	82 (93.2)
Month 6	88 (98.9)	88 (98.9)

Note: No significant difference between treatment groups

## Discussion

This study evaluated the efficacy of combined OCs containing EE/CMA and EE/DRSP for the treatment of mild to moderate acne vulgaris and dysmenorrhea. Our results showed more favorable benefits of using EE/CMA compared to EE/DRSP. Treatment with EE/CMA for 6 cycles showed a reduction in total acne lesions by 72.2% compared to the baseline level while 64.5% reduction was observed with EE/DRSP. This represents a 11.9% better improvement in acne treatment with EE/CMA over EE/DRSP (*p* = 0.009). This greater reduction in total acne lesion with EE/CMA was mainly from reductions in comedones and papules, as shown in Table 2, Fig. 2b and c. This result was consistent with previous EE/CMA phase III studies, showing 60–70% improvement in acne after 6 cycles of treatment [5, 6]. Moreover, it confirmed the result from a previous observational study that EE/CMA was significantly more beneficial compared to EE/DRSP [13]. The difference in the benefits on acne treatment between the two groups may be, in part, due to the unique property of each progestogen when combined with EE.

From the investigator’s global assessment on efficacy of treatment for acne vulgaris, markedly significant improvement graded as “excellent” response was observed in more proportion of subjects treated with EE/CMA (75.3%) than EE/DRSP (49.4%). When considering the subjects’ self-assessment on efficacy, similar responses with those of the investigator’s assessment was seen. Higher proportion of subjects treated with EE/CMA (66.3%) graded their improvement in acne as “excellent” than those treated with EE/DRSP (48.3%). The greater “excellent” response rating on efficacy of acne treatment in both investigator’s and subjects’ assessment with EE/CMA over EE/DRSP was consistent with the primary outcome showing statistically greater reduction of total acne lesion counts observed and therefore, reflected a clinically significant efficacy of EE/CMA in acne treatment. Moreover, this is consistent with the subjects’ self-assessment on intention to continue treatment. More subjects treated with EE/CMA would continue treatment compared to those from the EE/DRSP (83.1% vs 49.4%). This finding may influence the women’s treatment compliance to the OC in general.

Dysmenorrhea is one of the most commonly reported menstrual disorder and frequent complaint in women. Although symptoms are usually not serious and typically last within a few days, dysmenorrhea can be severe enough to have a significant impact on daily life functioning, causing work or school absenteeism. In this study, all of the subjects enrolled were women who were suffering with mild to moderate dysmenorrhea. Nearly half (47.2%) of the subjects treated with EE/CMA in this study reported an absence of dysmenorrheic pain as early as 1 month post-treatment while only 27.3% of the subjects treated with EE/DRSP reported an absence of the symptom. After 6 cycles of treatment, the symptom was completely absent in 95.5% of the subjects in the EE/CMA whereas 85.4% of the subjects in the EE/DRSP reported lack of dysmenorrhea. The absence of dysmenorrhea in the EE/CMA group in our study was higher compared to a previous report which had only 66% [6]. EE/CMA is not only effective in reducing dysmenorrhea but is rapid acting. Women with dysmenorrhea on EE/CMA may have fewer absenteeism from work or school. One possible explanation for the significant difference in the effect between EE/CMA and EE/DRSP has been postulated to be due to the progestogen component. CMA is speculated to have a special pharmacological action in relation to endometrial arachidonic acid metabolism. It binds to glucocorticoid receptor which may inhibit phospholipase A2, and this, combined with inhibition of cyclo-oxygenases, results in reduction of prostaglandin levels [7]. This consequently may lead to a superior benefit of CMA in improvement of dysmenorrhea.

EE/CMA and EE/DRSP were both generally safe and well-tolerated. Most frequent adverse events during the 6 cycles of treatment were breast pain, dizziness, acne, headache, and nausea. The incidence rates of these events were similar between EE/CMA and EE/DRSP. These adverse events are commonly known OC-associated events. Moreover, no significant weight change was detected in both treatment groups. Previous post-marketing surveillance study has shown the benefits of using EE/CMA on bleeding disorders and cycle control [6]. In this study, after 6 cycles of treatment, both OCs provided good cycle control but there were fewer breakthrough bleeding among subjects using EE/CMA.

The strengths of this present study are that it was a randomized controlled trial which prevented selection bias and had an adequate sample size to determine the statistical significance of the primary study endpoint. The important limitation of this study is its single-blinded methodology which may be affected by some bias in the subjects’ self-assessment.

## Conclusions

This study demonstrated that EE/CMA is significantly more effective for the treatment of acne and dysmenorrhea in women with mild to moderate acne vulgaris and dysmenorrhea than EE/DRSP. Our results confirm the beneficial effects of EE/CMA over EE/DRSP which were reported previously. These non-contraceptive health benefits could influence the women’s choice of OCs and better adherence to treatment.

## Abbreviations

CMA: Chlormadinone acetate
COCs: Combined oral contraceptives
DRSP: Drospirenone
EE: Ethinyl estradiol
EE/CMA: Ethinyl estradiol/Chlormadinone acetate
EE/DRSP: Ethinyl estradiol/Drospirenone
ITT: Intention-to-treat
OCs: Oral contraceptives
PP: Per-protocol

## References

[1] Rabe T, Runnebaum B (1999). The future of oral hormonal contraception: fertility control — update and trends.

[2] Schindler AE (2013). Non-contraceptive benefits of oral hormonal contraceptives. Int J Endocrinol Metab.

[3] Wong CL, Farquhar C, Roberts H, Proctor M (2009). Oral contraceptive pill for primary dysmenorrhoea. Cochrane Database Syst Rev.

[4] Zahradnik HP, Goldberg J, Andreas JO (1998). Efficacy and safety of the new antiandrogenic oral contraceptive Belara. Contraception.

[5] Worret I, Arp W, Zahradnik HP, Andreas JO, Binder N (2001). Acne resolution rates: results of a single-blind, randomized, controlled, parallel phase III trial with EE/CMA (Belara) and EE/LNG (Microgynon). Dermatology.

[6] Schramm G, Steffens D (2003). A 12-month evaluation of the CMA-containing oral contraceptive Belara: efficacy, tolerability and anti-androgenic properties. Contraception.

[7] Zahradnik HP (2005). Belara--a reliable oral contraceptive with additional benefits for health and efficacy in dysmenorrhoea. Eur J Contracept Reprod Health Care.

[8] Ardila MD, Binek M, Mojica C, Sanchez F (2005). Experiences with the new oral contraceptive EE/CMA (ethinyl estradiol/chlormadinone acetate) in Columbia: an observational phase IV study.

[9] van Vloten WA, Van Haselen CW, van Zuureen EJ, Gerlinger C, Heithecker R (2002). The effect of 2 combined oral contraceptives containing either drospirenone or cyproterone acetate on acne and seborrhea. Cutis.

[10] Harada T, Momoeda M, Taketani Y, Hoshiai H, Terakawa N (2008). Low-dose oral contraceptive pill for dysmenorrhea associated with endometriosis: a placebo-controlled, double-blind, randomized trial. Fertil Steril.

[11] Momoeda M, Kondo M, Elliesen J, Yasuda M, Yamamoto S, Harada T (2017). Efficacy and safety of a flexible extended regimen of ethinylestradiol/drospirenone for the treatment of dysmenorrhea: a multicenter, randomized, open-label, active-controlled study. Int J Womens Health.

[12] Strowitzki T, Kirsch B, Elliesen J (2012). Efficacy of ethinylestradiol 20 mug/drospirenone 3 mg in a flexible extended regimen in women with moderate-to-severe primary dysmenorrhoea: an open-label, multicentre, randomised, controlled study. J Fam Plann Reprod Health Care.

[13] Sabatini R, Orsini G, Cagiano R, Loverro G (2007). Noncontraceptive benefits of two combined oral contraceptives with antiandrogenic properties among adolescents. Contraception.

